# Cannibalism—overview and medicolegal issues

**DOI:** 10.1007/s12024-023-00623-4

**Published:** 2023-04-14

**Authors:** Roger W. Byard

**Affiliations:** 1 Forensic Science SA, 5000 Adelaide, SA Australia; 2 School of Biomedicine, The University of Adelaide, Level 2, Room N237, Helen Mayo North, Frome Road, 5005 Adelaide, SA Australia

**Keywords:** Cannibalism, Serial murder, Sexual murder, Ritual, Sadism

## Abstract

Cannibalism, the consumption of another by an individual of the same species, is a widespread practice amongst many animal groups. Human cannibalism or anthropophagy, however, is less common but has been found in many diverse groups ranging from hominids to Crusaders and soldiers in World War II. Although the existence of human cannibalism has been vigorously debated in recent times, it seems clear that well-described cases have occurred. The motivation for consuming human tissues may be (1) nutritional, (2) ritual and (3) pathological. A case of alleged cannibalism involving one of the victims of the so-called Snowtown serial killings in South Australia, Australia, is reported with an analysis of the history and features of cannibalism. Forensic problems may occur in accurately identifying remains that have been cannibalized; however, if ritualistic, serial and/or sadistic homicides are encountered, cannibalism should be considered, particularly if body parts are missing.

## Introduction

Cannibalism refers to the consumption of another by an individual of the same species. The word cannibal derives from *Caníbales*, the Spanish name for the Caribe Indians of the Lesser Antilles who were believed to indulge in this practice [1]. It is widespread in many animal groups, including protozoa, insects, fish, birds and mammals, where it is considered not aberrant behavior but a ‘normal response to many environmental factors’ [2].

Human cannibalism or anthropophagy, however, is far less common, although it has occurred for millennia in a wide range of human societies and cultural groups. Anthropological studies have identified cut marks on 780,000-year-old hominid bones and on 100,000-year-old bones from Neanderthal populations that existed in the Middle Paleolithic era [1, 3, 4]. According to the optimal foraging theory, it has been proposed that cannibalism in early hominid populations was driven by the ready availability of fellow hominids with respect to other food sources making them a ‘high-ranked prey type’ [5].

**Fig. 1:**
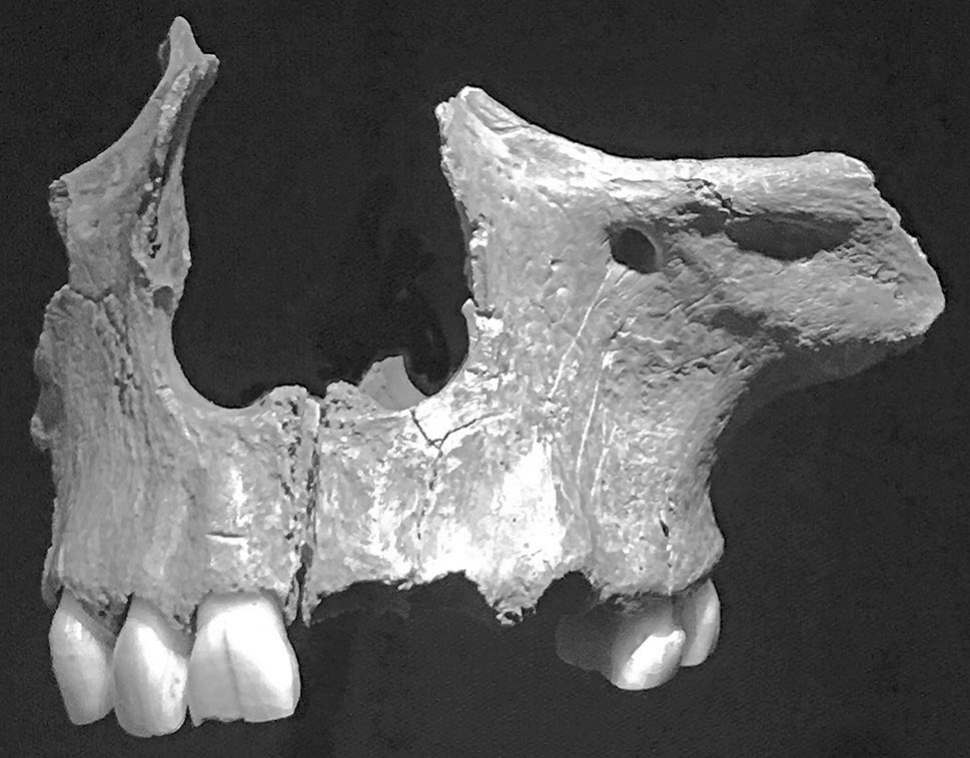
A portion of maxilla from Gough’s Cave showing cut marks suggestive of defleshing. (Natural History Museum, London, Nicolas Perrault III, made available under the Creative Commons CC0 1.0 Universal Public Domain Dedication)

Similar remains (Fig. 1) from Goughs Cave in Somerset, England, date from the Early Holocene to the Pleistocene eras [6]. More recently, the finding of broken bones with tooth marks and cuts, with signs of cooking, showed that it was occurring in Early Bronze Age Europe, just 4000 years ago [7]. Crusaders allegedly cannibalized their dead opponents during the Siege of Ma`arra in the First Crusade (1095–99) and possibly on the march to Jerusalem [8].

Characteristic features of cannibalism in archeological material include the finding of admixed fragments of human and animal bones, all showing similar features with typical signs of butchery, often alongside stone implements [1]. The bones are generally fragmented with cut marks associated with the removal of attached tendons and soft tissues, in addition to peeling fractures from the bending of long bones and percussion and chop marks from smashing of bones to extract marrow [1]. The finding of complete ribs with only fragmented limb bones is supportive evidence for this [9]. Features such as these may also be used in more contemporary skeletonized cases to indicate that cannibalism has occurred (see below). Although it has been suggested that similar bone trauma may be found in cases of second burials where bones have been cleaned before reburial, this has been disputed as the pattern of bone trauma is different [9].

**Fig. 2:**
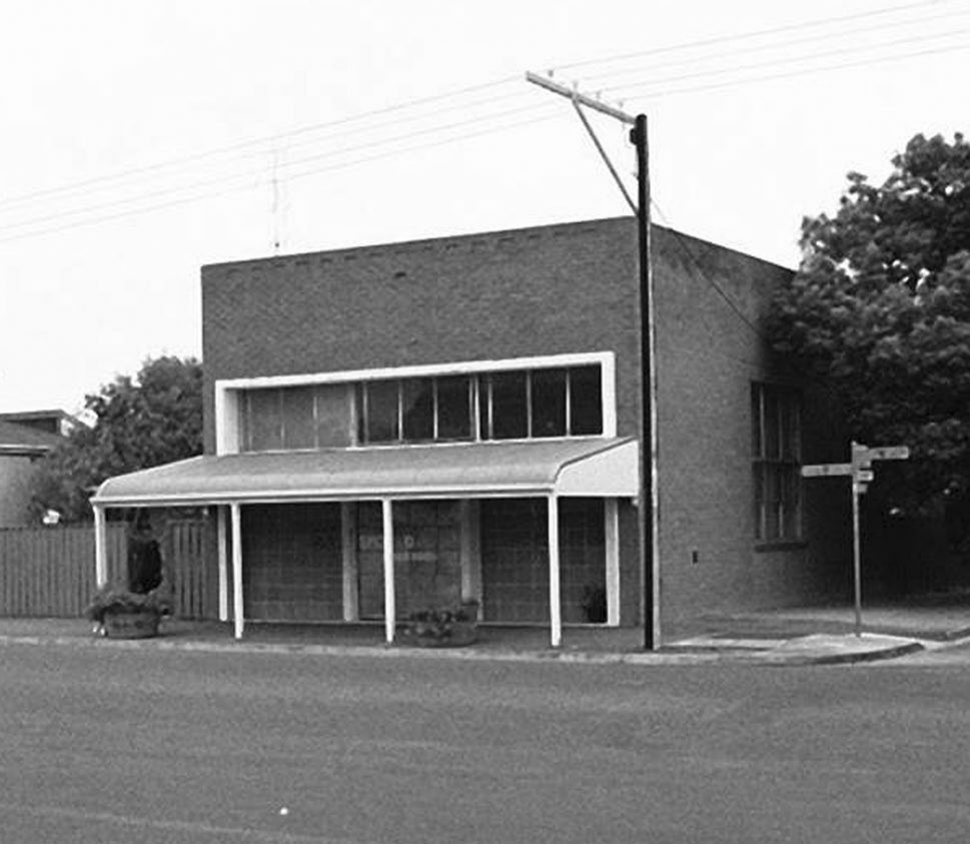
The front of the bank in Snowtown, South Australia, where six barrels were found in May 1998 containing eight intact and partially dismembered bodies

While cannibalism may simply take place for nutritional purposes, there are also cases that occur for ritual and pathological reasons. During the investigation of a series of bodies found stored in large plastic barrels in the vault of a disused bank in the South Australian village of Snowtown (Fig. 2), a description of cannibalism was given by one of the perpetrators. This case of alleged cannibalism is described as an introduction to an analysis of the types of cannibalism that may be encountered in a forensic context, with specific medicolegal issues that may arise.

## Case report

Following a detailed police investigation, six barrels that were discovered in the vault of a bank in Snowtown, South Australia, in May 1998 were transported to the Forensic Science Centre in Adelaide where a team led by pathologists John Gilbert and Roger Byard examined the contents. The barrels contained eight intact and partially dismembered adult bodies consisting of seven males and one female, along with hand cuffs, thumb cuffs, disposable gloves, duct tape, gags and rope. At the end of the investigation, it transpired that there were 12 victims in all, with 4 bodies being disposed of at other sites. The causes of death were often difficult to determine given the state of decomposition of the bodies, with a number of pathological conclusions of ‘undetermined’ being made; however, there was evidence of strangulation, choking and drug ingestion in several of the victims. The killings had taken place between 1992 and 1999 with John Bunting, Robert Wagner and James Vlassakis subsequently being convicted of multiple counts of murder [10].

During the trial, it was asserted by Vlassakis that Wagner had removed flesh from the final victim, David Johnson which was then cooked in a frying pan and eaten [10, 11]. He claimed that the tissue came from the right thigh of the deceased and this was supported by the autopsy examination which had revealed an 18 × 18 cm defect where a piece of skin and muscle had been removed from the medial aspect of the right lower thigh (although a fragment of skin and muscle that could match the defect was also present with the body in the barrel) (This illustrates difficulties that may arise in relying upon pathological examinations to assess soft tissue injuries in the presence of decomposition).

## Discussion

Determining whether cannibalism has occurred may not be possible based on autopsy findings, as was demonstrated in the Snowtown case where only a tissue defect that could correspond to a portion removed for consumption was found. As the early stages of dismemberment consist of taking slices of thigh and/or buttock muscle, this could be obscured by subsequent putrefactive changes. This has been termed ‘survival cannibalism’. Later, more intensive searching for nutritive material, ‘end stage cannibalism’, may leave detectable markings such as cut marks on the bone (Fig. 1) or the breaking of long bones to access marrow with hammer stone abrasions. Long bones that have been boiled may show characteristic end polishing of their ends from rubbing against a pot side during cooking [12, 13].

### Incidence

It has been suggested based on animal studies that the transmission of pathogens from victims to consumers may account for the rarity of cannibalism in human populations [14]; however, ethnographic and sociological factors are probably of more importance. The presence of certain genetic polymorphisms in human populations has been proposed as evidence of an evolutionary protective mechanism against kuru-type diseases associated with cannibalism (see below) possibly indicating that cannibalism may have been more common during earlier stages of human evolution [15, 16].

### Classifications

While the existence of cannibalism has been disputed, particularly in tribal societies, there appears no doubt from multiple historical records that there have been many reliably reported cases. Cannibalism occurs for a wide variety of reasons and may be separated into two broad categories of exo- and endocannibalism [9]. With exocannibalism, the victim is selected from outside the social group or community that is engaging in anthrophagy. The reasons for this may simply be nutritional in that outsiders are being hunted as a source of food, or tribal in that cannibalistic rituals form part of victory celebrations in warfare. The purpose of the latter is often to demonstrate complete dominance over a fallen foe and also to subjugate or incorporate their spirit into self [9]. Endocannibalism refers to cases where victims derive from the same social group and may be subclassified as ‘aggressive’ when the victim is an enemy or ‘affectionate’ when family or friends are involved. Again, the motive may be simply nutritional or ritual with domination and/or incorporation of the victim into the perpetrators [1].

A more functional classification is based on motivations for consuming human tissues and has the following subgroups: (1) nutritional or gastronomic, (2) ritual and (3) pathological [1]. Nutritional cannibalism has a long history and it has been hypothesized that early hominids engaged in cannibalism as fellow group members were readily available for consumption [5] and also that it served to dispose of corpses thus decreasing the attention of animal predators around camps. The basis of nutritional cannibalism is simply the use of human tissues or organs for their calorific value. It most often occurs in situations of acute starvation when groups are deprived of food or become isolated from their usual food resources.

### Historical examples

Historical examples of nutritional cannibalism have included survivors of shipwrecks who either fed on the bodies of those who had died, so-called necro-cannibalism, or who killed other survivors for food (homicidal cannibalism). A well-known example is the French frigate Méduse that sank off Mauritania in 1816. Of the 147 passengers and crew who were on a raft, only 15 survived. The Donner party of settlers trying to cross the Sierra Nevada mountains in the 1840s are another example of cannibalism in extremis [9].

**Fig. 3:**
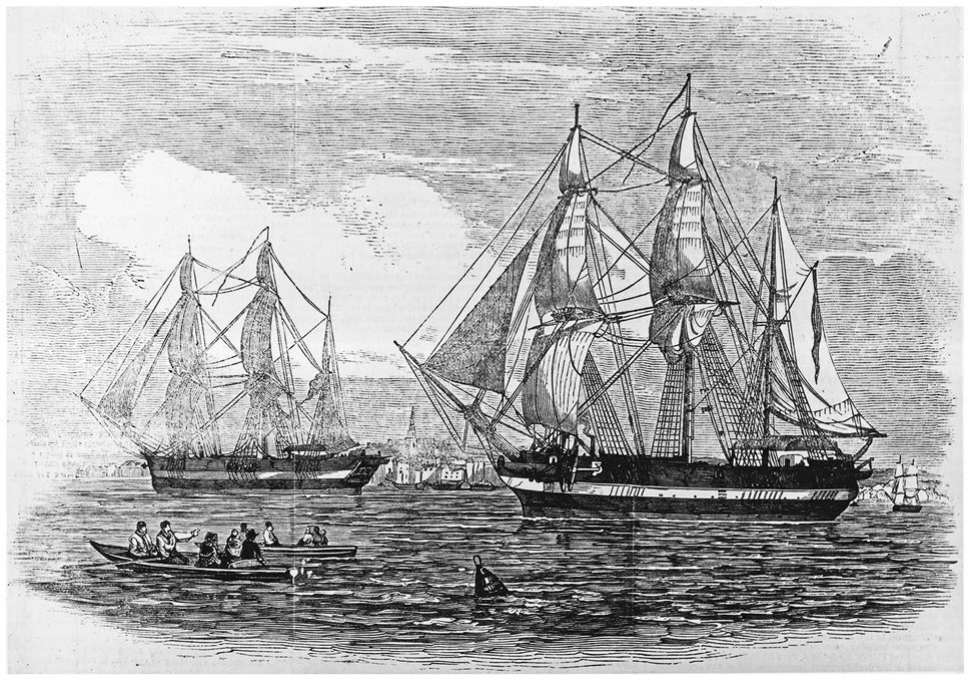
The departure of the “Erebus” and “Terror” in 1845 on the ill-fated Franklin Arctic Expedition (Illustrated London News -Public domain)

**Fig. 4:**
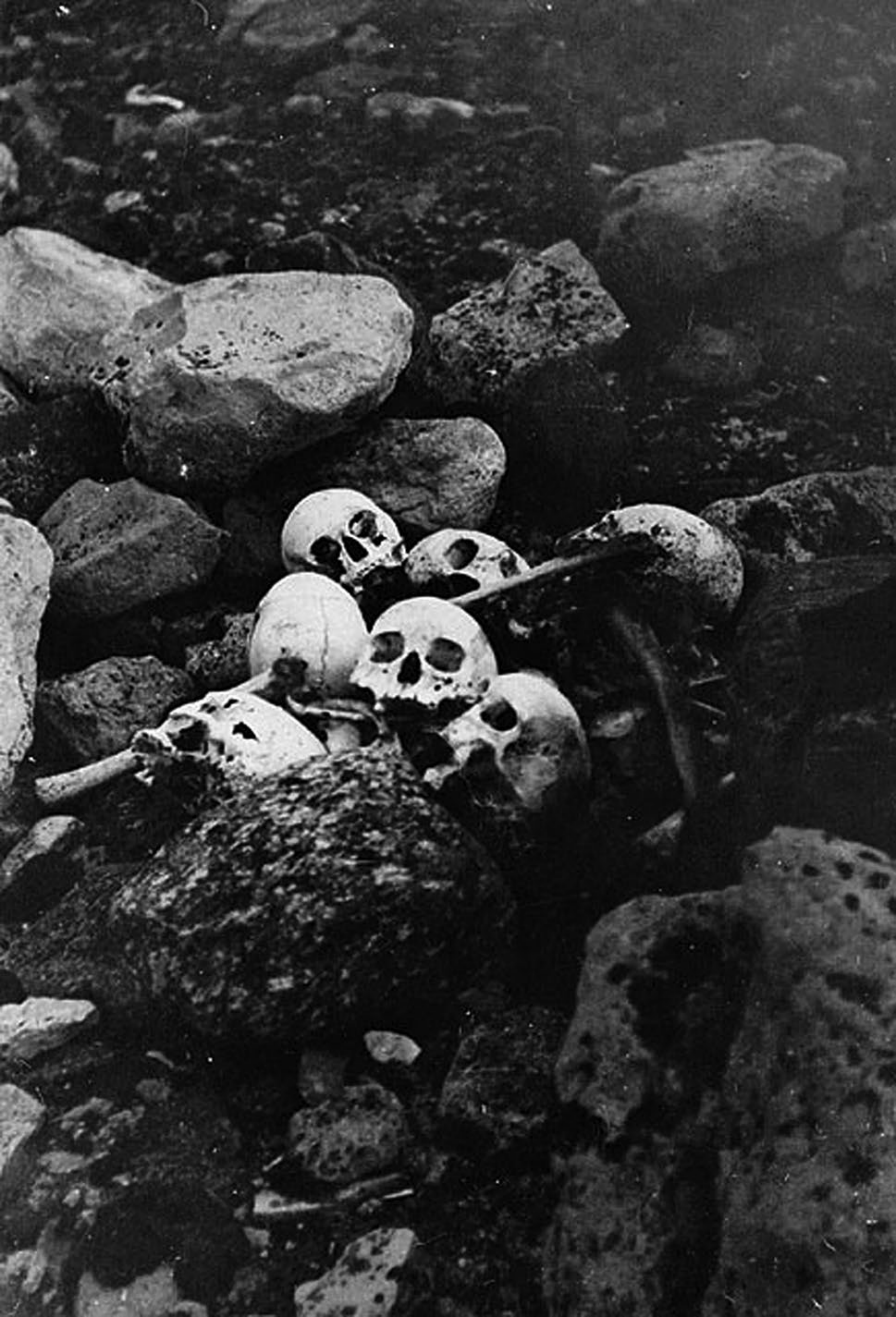
Skulls of sailors from the Franklin expedition found on King William Island (Library and Archives Canada)

The fate of the members of the Franklin expedition trying to discover the North-West passage in 1845 has been argued, with a number of proposed theories ranging from lead poisoning from soldered tins of preserved food to botulism and scurvy [17]. There appears no doubt, however, that the men engaged in nutritional cannibalism in the face of dwindling food supplies with recovered bones showing cuts and ‘pot polishing’ [13] (Figs. 3 and 4).

Nutritional cannibalism by necessity may progress to cannibalism by choice as was demonstrated by the nineteenth-century convict, Alexander Pearce who was imprisoned on Sarah Island on the west coast of the colony of Van Diemen’s Land. He survived during his escape with a group of fellow convicts in 1821 by resorting to killing and eating the others. Following his return to the island, he again escaped and murdered and consumed parts of his fellow escapee prior to recapture, putting him into the rare category of a serial opportunistic cannibal [18].

### Medicinal cannibalism

On occasion, the consumption of human tissues and blood has been promoted for medical purposes. In Europe, particularly in the sixteenth and seventeenth centuries, remedies were dispensed that contained human, blood, fat and bones derived from recent graves or from Egyptian mummies [19]. The ‘Kings Drops’ taken by Charles II contained human skull bones in alcohol, and moss growing over a buried skull was used to treat epilepsy and nosebleeds. Paracelsus considered that drinking blood was therapeutic [20].

The Encyclopaedia Brittannica of 1797 specified that ‘We have two different fubftances preferved for medicinal ufe under the name of mummy. . . The one is the dried and preferved flefh of human bodies, embalmed with myrrh and fpices; the other is the liquor running from fuch mummies’ [21].

### Ethnographic examples

**Fig. 5:**
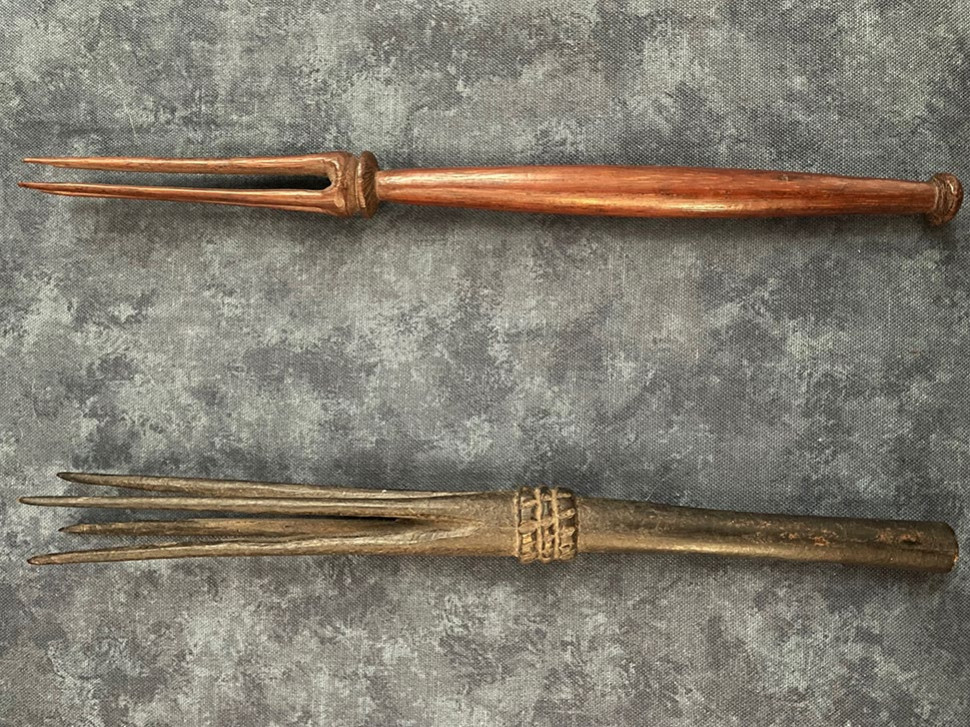
Traditional cannibal forks, or *bulutoko*, that were used by priests and chieftains in Fiji for eating human flesh so that they did not have to touch it

Although cannibalism was documented amongst a wide range of tribal groups such as the Hurons and Iroquois of North America, the Ashanti of West Africa and the Maori of New Zealand [22], it was suggested by Arens in 1979 that this was based on misrepresentations by early Western observers who consisted of biased missionaries and explorers striving to marginalize conquered peoples and assert cultural superiority [23]. This position has, however, been criticized with examples from Fiji used to refute it. Specifically, human skeletal remains from Viti Levu in Fiji have shown characteristic features of burning and crushing with cut marks. These findings were also corroborated by direct observations documenting this activity in the early part of the nineteenth century [24]. So-called cannibal forks, or *bulutoko*, were used by priests and chieftains in Fiji for eating human flesh (Fig. 5).

Certainly, a well-documented culture of endocannibalism occurred amongst the South Fore people of the Eastern Highlands of Papua New Guinea which resulted in the spread of Kuru a prion-based spongiform encephalopathy which led to fatal dementia [16, 25]. Between 1957 and 1961, there were approximately 1000 kuru-related deaths in the area [26].

### Recent examples

The crash of Uruguayan Air Force flight 571 in the Andes in 1972 left a group of passengers including members of a school football team stranded at high altitude without food. They were rescued after 72 days but had only been able to survive by resorting to feeding on the dead. The story has been the subject of a book, film and documentary [22, 27].

### Famine-induced cannibalism

**Fig. 6:**
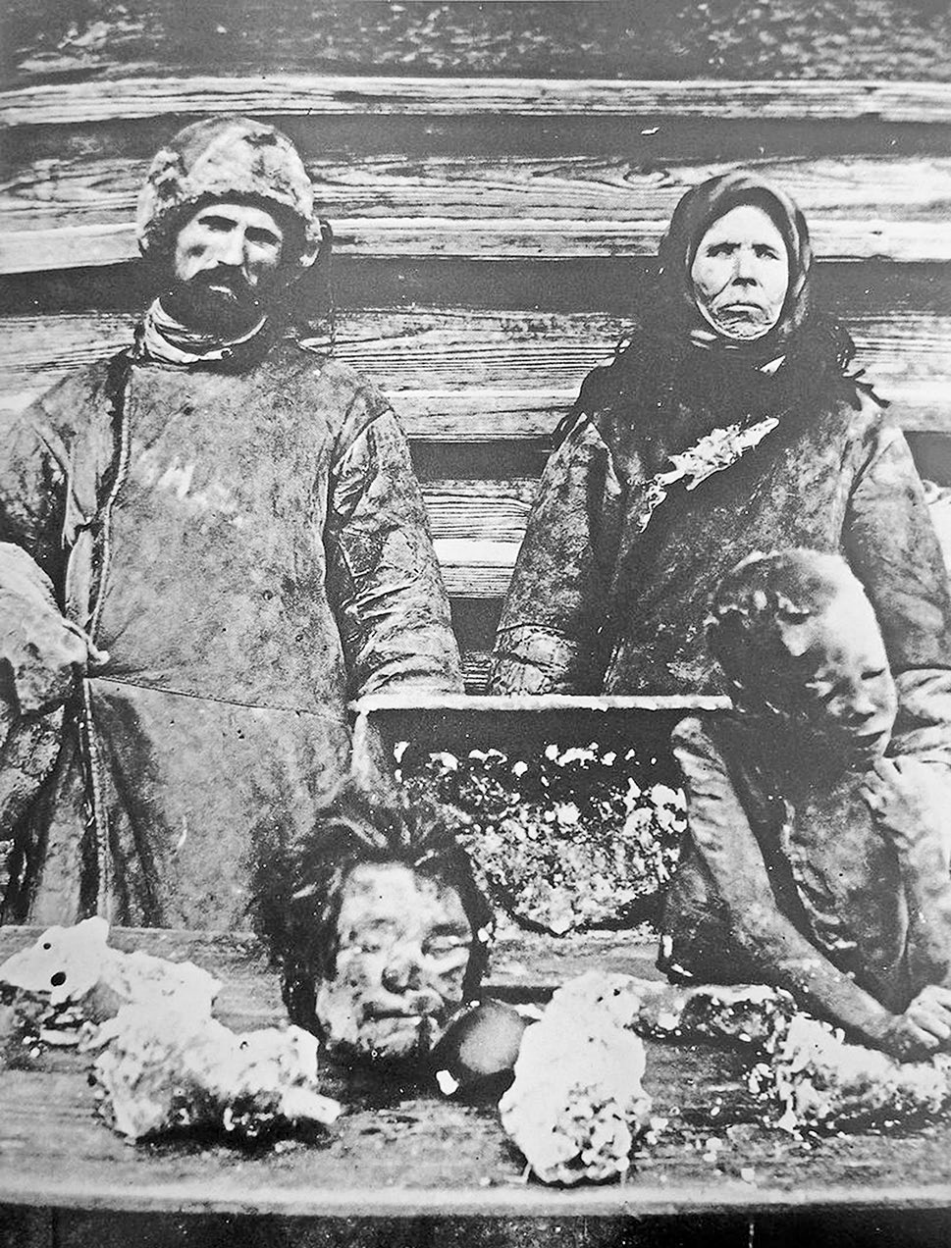
Cannibals with portions of their victims in the Samara province of the Volga region during the Russian famine of 1921–1922 (Public domain)

Although sometimes disputed, a number of major famines in the twentieth century have been associated with cannibalism including Russia from 1921 to 1922 (Fig. 6) and in Ukraine after Stalin’s collectivisation from 1929 to 1933 (22) where over 2000 people were subsequently sentenced for engaging in the practice. German prisoners of war resorted to cannibalism after the fall of Stalingrad in World War II, where human tissue was referred to as ‘camel meat’ [28], and it was documented to have occurred in German concentration camps [29]. Japanese troops after World War II were found guilty of eating both prisoners of war and civilians [30]. There was evidence of cannibalism in records from famines in Moldova in the 1940s and in China during the Great Leap Forward of 1959–1961 [30].

### Ritual cannibalism

Ritual cannibalism occurs in tribal groups where belief systems or religions may require the sacrifice and ingestion of a victim, or the consumption of the already dead. While the early Spanish accounts of Aztec sacrifices followed by cannibalism have been criticized for ethnocentric bias, it appears clear that human sacrifice with distribution of flesh for eating occurred. In fact, these activities involved a special group of old men who were called Quaquaquilti [9].

### Serial killers and pathological cannibalism

Consumption of their victims by killers is a recognised, although rare, occurrence with one of the most widely publicized being Jeffery Dahmer a serial killer in the USA who committed 17 murders between 1978 and 1991. He was found to have engaged in necrophilia, consuming parts of his victims and keeping skulls and body parts as souvenirs [9, 31]. A slightly different case was that of Armin Meiwes in Germany who advertised on the internet for a victim to kill and consume [32]. Although unproven, the possibility of cannibalism occurring in the setting of the sadistic Snowtown murders would not, therefore, be surprising [33, 34].

Individuals who engage in such practices are usually either severely mentally ill or suffering from a significant paraphilia [35, 36]. The same applies to those who suffer from clinical vampirism where they drink human blood, usually from a deceased or dying victim [37]. A review of cannibalistic homicides showed that the offenders were older and the victims younger than in other homicides, that deaths were inflicted manually (beating, strangling and stabbing) rather than by gunshots and that kin-avoidance occurred with more victims being strangers. When family members were victims, the perpetrators were usually suffering from more serious psychiatric illnesses [38].

### Self cannibalism (autosarcophagy)

While cases of self-consumption are very rare and are associated with psychosis [39], it has been documented in the absence of severe psychiatric disease and substance abuse [40–42]. As the placenta is actually part of the infant, placentophagy (postpartum eating of the placenta) [43] is more correctly classified as cannibalism and not autosarcophagy. This is a practice that is becoming increasingly common particularly in the USA where placentas may be eaten raw or roasted, dehydrated or consumed in smoothies [43].

### Medicolegal issues

The identification of cannibal activity from contemporary skeletonized remains relies upon finding similar features to those found in archaeological sites of cuts to the bone and trauma to long bones, in addition to signs of cooking such as pot polishing. Soft tissue defects such as the one identified in the reported Snowtown case may be more difficult to assess if there has been putrefaction and decomposition with loss of tissue morphology. In cases such as Jeffrey Dahmer, the preservation of organs for consumption in a freezer may simplify the evaluation [9]. In cases where corpse dismemberment has occurred, it may be again difficult to determine the precise fate of the missing/limbs/organs.

A legal issue may be whether cannibalism occurred after death (necrocannibalism) or whether instead the victim was killed prior to consumption (homicidal cannibalism). Removal of tissues or limbs prior to death may be associated with bruising and hemorrhage. Harvesting flesh from prisoners while they were alive by Japanese soldiers in World War II led to convictions in trials after the war [44].

The ‘rule of the sea’ was sometimes evoked to excuse shipwrecked sailors who had decided to sacrifice victims and resort to cannibalism. In 1884 when the yacht Mignonette sank off the Cape of Good Hope, two of the four survivors in a life raft decided to kill a cabin boy [45]. After rescue, they were charged with murder with the conclusion of the court being that ‘necessity is no defence to a charge of murder at law even where the murder was committed to enable the survival of others’ [46].

## Conclusion

Thus, although cannibalism is now rare in most Western societies, it has had an extensive history occurring in many communities and cultures over millennia. Its association with sexual serial murders [32], however, means that it may be something to consider if ritualistic, serial and/or sadistic homicides are encountered, particularly if body parts are missing.
